# The Association of Anti-Ganglioside Antibodies in the Pathogenesis and Development of Zika-Associated Guillain-Barré Syndrome

**DOI:** 10.7759/cureus.8983

**Published:** 2020-07-03

**Authors:** Divya Mohite, Janet A Omole, Karandeep S Bhatti, Thanmai Kaleru, Safeera Khan

**Affiliations:** 1 Neurology, California Institute of Behavioral Neurosciences and Psychology, Fairfield, USA; 2 Internal Medicine, California Institute of Behavioral Neurosciences and Psychology, Fairfield, USA

**Keywords:** zika virus, gbs, anti-ganglioside antibodies, paralysis, molecular mimicry

## Abstract

Zika virus (ZIKV) has created major outbreaks all over the Americas and has caused severe neurological complications. The main neurological complications linked to ZIKV are Guillain-Barré syndrome (GBS), encephalitis, myelitis, and microcephaly. We thoroughly searched for published literature on PubMed and found evidence supporting the relationship between ZIKV and GBS. Through April 1, 2020, 429 publications were available on PubMed using the words “Zika associated GBS.” Among these, only four results linked anti-ganglioside antibodies to Zika-associated GBS. So, we expanded our search to other platforms like PubMed Central® (PMC), Google Scholar, and Cochrane, after which we shortlisted 28 studies. These studies include review articles, observational studies, case series, and case reports. The information collected from these articles were mainly based on the outbreaks in Latin America and the results that these patients showed in the course of the disease. It took a lag time of 7-10 days for the patients to develop Zika-associated GBS. We used all the evidence regarding the epidemiology, clinical manifestations, neurological complications, and diagnostic criteria that supported the findings of anti-ganglioside antibodies to ZIKV-associated GBS. Patients were detected with the presence of these antibodies in their urine through the enzyme-linked immunosorbent assay (ELISA) test. But the mechanism by which the ZIKV causes other complications like myelitis and encephalitis is still unknown and yet to be explored to develop treatment and management strategies.

## Introduction and background

Zika virus (ZIKV) belongs to the family of Flaviviridae, which is transmitted by the Aedes mosquito (arthropod-borne) [1]. It can be transmitted sexually, in-utero and its presence is even noted in breast milk [2]. The first outbreak of this virus was in Micronesia in 2007, followed by 2014 and 2015 outbreaks in French Polynesia and Latin America, respectively. An increasing number of Guillain-Barré syndrome (GBS) cases were noted in these patients [1,3,4]. Zika is a neurotropic virus that is neurovirulent but does not possess a neuro-invasive character. Hence, it can cause microcephaly in human fetuses (intrauterine infection) and GBS in adults [5]. Microcephaly cases mainly affect pregnant women in their first trimester [6]. In a study conducted, the absence of ZIKV was found in nervous tissue, which clearly showed that the pathogenesis of ZIKV-associated GBS is antibody-mediated rather than neurotropic [7].

GBS is among the most common autoimmune polyneuropathies with a post-infectious etiology. It is a form of ascending paralysis, which is rapidly progressive and causes symmetric weakness of the extremities [8]. The pathogenesis of GBS is said to be molecular mimicry between the gangliosides and the molecules present on the surface of the infectious agents (e.g., lipopolysaccharide of Campylobacter jejuni). Autoimmunity between these gangliosides and ZIKV is what contributes to the neurological complications of this virus [9]. Not all patients infected with Zika develop neurological complications [10]. In a case-control study comprising 29 patients with ZIKV-associated GBS and 74 control patients with solely Zika infection, all the GBS patients were positive for anti-Zika IgG antibodies. The lag time between this viral infection and neurological symptoms was seven days [11]. Areas of brain tissue softening, neuronal degeneration, and inclusion bodies were noted in Swiss albino mice of all ages in a mouse model when intracerebral inoculation of a strain of ZIKV (E/1 - isolated from Australopithecus africanus) was conducted. They demonstrated hind limb paralysis, and increased levels of ZIKV RNA were noted in the brain and spinal cord [12]. 

**Figure 1:**
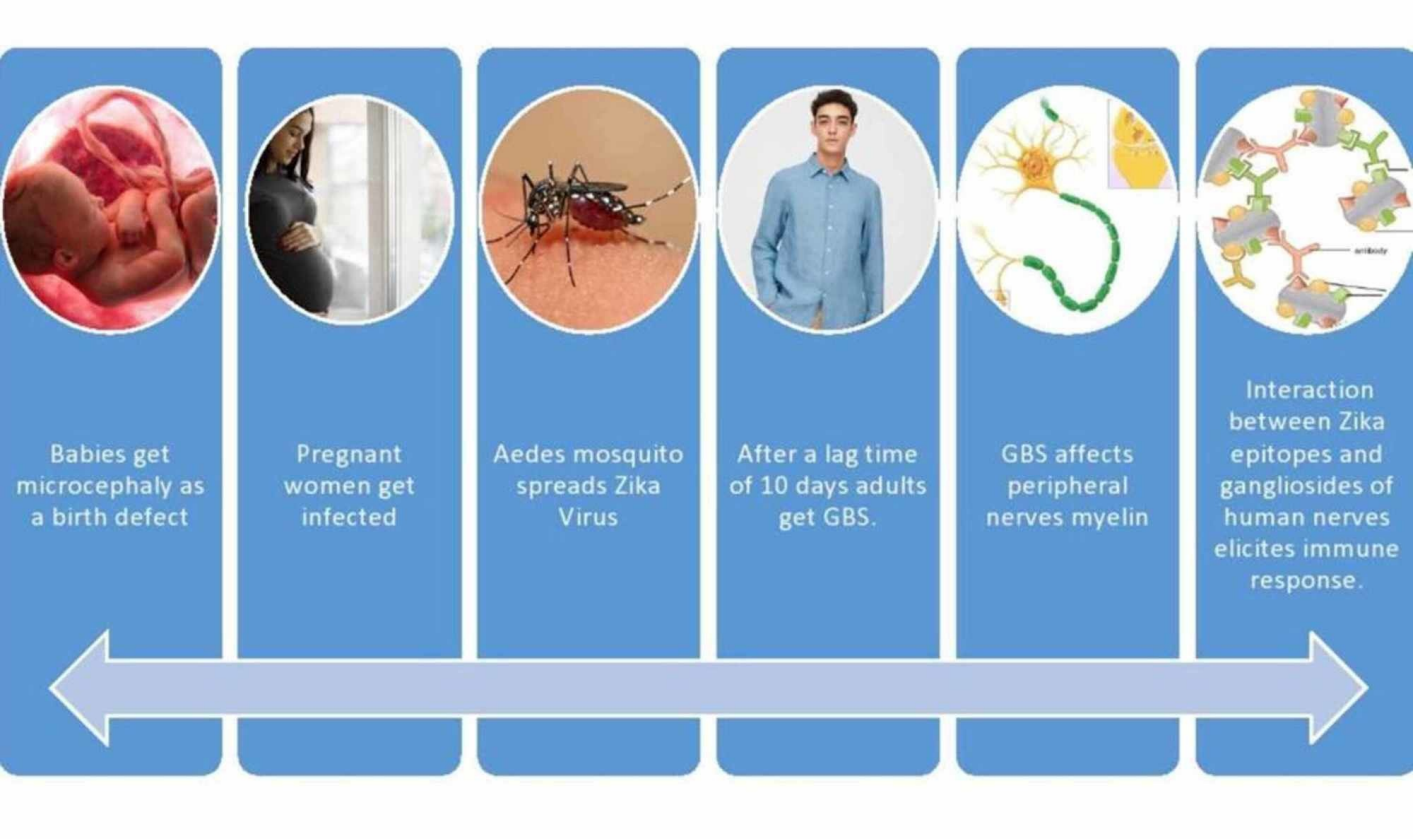
This image illustrates the development of GBS and microcephaly from the bite of the Aedes mosquito and the pathway by which it is transmitted to people. GBS: Guillain-Barré syndrome.

The presence of anti-ganglioside antibodies was found in patients infected with pathogens like C. jejuni, Epstein-Barr virus, and cytomegalovirus [9]. However, the association of anti-ganglioside antibodies in patients with Zika-associated GBS is still unclear. Postmortem investigation of post-infectious GBS patients can give us an insight into the molecular mechanism [7]. Gangliosides are a type of glycosphingolipids that contain a ceramide lipid anchor and sialic acids attached to a neutral sugar backbone [13]. They play a crucial role in neurogenesis and synaptogenesis and are required for the development of human neuronal progenitor cells [10]. Hence, the autoimmune response that causes damage to these gangliosides can lead to serious neurological complications like GBS. In this study, we will be focusing on the association between anti-ganglioside antibodies in patients with Zika infection complicated by GBS and the mechanism by which they occur. Figure 1 explains the pathway by which ZIKV causes microcephaly and GBS.

## Review

Methods

We searched for the ZIKV-related articles published up until 2020 in public electronic databases, including PubMed, PubMed Central® (PMC), and Google Scholar. The search words used were “Zika associated GBS” and “anti-ganglioside antibodies.” After a thorough literature search, we selected 28 studies providing evidence on a link between ZIKV and neurological disorders such as GBS, myelitis, and encephalitis in adults and microcephaly in infants. We summarized the relevant studies as the evidence used to support the link for the neuropathogenesis of ZIKV and its association with GBS.

Results

As of April 1, 2020, Pubmed listed 429 results with the search word “Zika associated with GBS.” Then we narrowed our list by adding anti-ganglioside antibodies to our search, which gave us only four results. So, we expanded our search more rigorously by searching for the mechanism of neurological complications caused by the ZIKV, which gave us 62 more results. We also used PMC, Google Scholar, and Cochrane. After going through all these publications, we shortlisted 28 studies [1-28], which were the most closely associated with our topic of neuropathogenesis of Zika virus and autoimmune mechanism of Zika-associated GBS. Among these 28 studies, there were review articles (n=15), observational studies (n=6), animal model approach (n=5), and case series (n=2). The observational studies mainly focused on the detection of anti-ganglioside antibodies using enzyme-linked immunosorbent assay (ELISA) test in those who developed GBS with a recent history of ZIKV and these animal model approaches were used to give us an insight on the pathogenesis and molecular theory of GBS development. The presented case series was published during the outbreaks in 2016 in Latin America.

Discussion

Neuropathogenetic Pathway of ZIKV Complications

ZIKV is an RNA flavivirus that causes Zika fever. Its symptoms last for seven days and can cause joint pain, headache, fever, and conjunctivitis, which is similar to other Flaviviridae like dengue. The past outbreaks of this virus have caused a myriad of neurological complications such as GBS, congenital Zika syndrome (microcephaly), meningoencephalitis, and myelitis [14]. In 2016, a case series was conducted by Arias et al. in Cúcuta, Columbia, which included 19 patients in their study. All these patients had GBS and a recent history of ZIKV infection. It took 10 days for these patients to develop neurological complications [15]. Whereas, Barbie et al. did a systematic review, which showed very low occurrences of GBS cases in Zika-infected patients [16]. This result was opposed by Nascimento and da Silva in 2017. After going through several case reports and case series, they concluded that the ZIKV outbreak brings alongside a bundle of complications, especially GBS and its variants [17]. It was proved that this virus causes several complications, but the mechanistic insight into the pathogenesis by which this virus causes these neurological defects was still unknown.

The virus uses three mechanisms to cause neurological infection/complications in humans. These are neuroinvasiveness, which enables the virus to enter the central nervous system (CNS), neurotropism that allows the virus to infect neuronal cells, and neurovirulence that gives the ability to the virus to cause CNS disease [5]. Highlighting this concept, in 2018, Mancera-Páez et al. postulated that the cases of GBS were para-infectious in origin, which was induced by the neurotropic effects of Zika to the peripheral nervous system (PNS) and CNS epitopes. This was indeed boosted by the existing passive immunity against arboviruses such as West Nile virus, chikungunya virus (CHIKV), or dengue virus (DENV) [18]. ZIKV infects the neural progenitor cells in humans, which causes microcephaly as a consequence of vertical transmission and musculoskeletal anomalies like GBS in adults [2,19,20]. The neurovirulence characteristic of this virus is still underexplored. Olagnier et al. showed that ZIKV infection disrupted the cell-cycle progression and caused cell death, which resulted in attenuated stem cell growth derived from human cortical progenitor cells [2]. These were the first clues that lead us towards the pathogenesis of congenital Zika infection. Consequently, Ikuso et al. proposed two mechanisms for the pathogenesis of ZIKV-associated complications - viral pathology in the brain, which causes microcephaly and immunopathology in the PNS, which causes GBS. Molecular mimicry between the microbes and gangliosides on the nerves is the mechanism by which post-infectious GBS is caused [5]. But the target of ZIKV in the PNS as ganglioside is still unknown. Autoimmunity between gangliosides and ZIKV epitopes is the mechanism by which GBS is caused, and direct viral toxicity of the neurons or glial cells is the cause of microcephaly and other neurological complications like encephalitis and myelitis [10,21].

There are many mechanisms hypothesized at a molecular level in the development of neurological complications by ZIKV. Piontkivska et al. postulated one of those. In 2019, that dysregulation of post‐transcriptional RNA editing can be one of the main drivers leading to neurological defects in both infants and adults. They collected evidence for ZIKV-mediated changes in the expression of adenosine deaminase on RNA, which let them find the link [22]. Between abnormal RNA editing and pathogenesis of Zika-associated neurological symptoms, Nayak et al. described another pathway that ZIKV activates toll-like receptor 3 in the brain, immune system as well as other organs like eye, skin, and male and female reproductive tracts, which describes one of the pathways by which this virus causes these complications [12]. Table 1 summarizes all the studies involved under this subheading which were used to explain the neuropathogenesis of Zika-associated complications. Molecular mimicry by neurotropism and direct viral toxicity of the neural progenitor cells are the mechanisms by which this virus causes GBS and microcephaly but the pathways by which ZIKV causes other neurological complications like transverse myelitis and meningoencephalitis are yet to be explored.

**Table 1:** The different studies used to explain the neuropathogenic pathway in the development of complications like microcephaly and GBS associated with the ZIKV. GBS: Guillain-Barré syndrome, ZIKV: Zika virus, CHIKV: chikungunya virus, DENV: dengue virus.

Author	Year of Publication	Purpose of the Study	Intervention Studied	Result/Conclusion
Piontkivska et al. [22]	2019	To show ZIKV pathogenesis as a result of host innate immunity.	The authors collect evidence of ZIKV‐mediated changes in the expression of adenosine deaminases acting on RNA.	Defect in post‐transcriptional RNA editing is the reason for observed neurodevelopmental defects and clinical symptoms in both infants and adults linked with ZIKV infections.
Mancera-Páez et al. [18]	2018	To explain the mechanism by which ZIKV causes neurological complications.	Studied case reports of different patients and followed them for the development of complications.	ZIKV is a neurotropic virus enhanced by passive immunity against other types of arboviruses like CHIKV or DENV.
Silva et al. [14]	2018	To review the concepts and clinical signs associated with complications of ZIKV infection.	Intravenous immunoglobulins have been used, as in conventional GBS.	Neurological complications are reported and include GBS and congenital ZIKV syndrome.
Barbi et al. [16]	2018	To review and perform a meta-analysis to estimate the prevalence of GBS among ZIKV-infected individuals.	They searched for studies published in international journals regarding ZIKV infection and its relationship with GBS.	A low estimate of GBS in ZIKV patients.
Tsunoda et al. [5]	2017	To explain the difference among neuroinvasiveness, tropism, and virulence of ZIKV.	To study the animal models and predict the route of ZIKV infection.	ZIKV is a neurotropic virus that infects the neural progenitor cells of the fetal brain. Anti-ZIKV antibodies were found in patients with GBS.
Russo and Beltrão-Braga [19]	2017	To review pathways of ZIKV-related neurological complications like microcephaly and GBS.	Data specifically suggest that neural progenitor cells are the main targets of ZIKV infection, causing massive cellular death and impairment in the neurogenesis process.	Vertical ZIKV infection causes microcephaly, and in adults, GBS and meningoencephalitis are noted.
Arias et al. [15]	2016	To present a case series of GBS possibly associated to ZIKV.	Collected clinical and demographic data from patients with GBS with a recent history of ZIKV in Cúcuta, Colombia.	All patients with GBS had a recent history of ZIKV, which proved the association between ZIKV and GBS.
Anaya et al. [10]	2016	To identify the risk factors associated with the neurological complications in ZIKV patients.	To understand the role of genetics, epigenetics, and the environment in the pathogenesis of autoimmunity in susceptible individuals.	The presence of gangliosides in patients with recent ZIKV infection proves the pathogenesis of Zika-associated GBS.
Li et al. [20]	2016	To discuss the neurobiology of ZIKV.	In vitro and in vivo models to study microcephaly and GBS.	ZIKV infection is linked to loss of fetal brain tissue, and the bias of stem cell populations in the adult brain supports this conclusion.
Olagnier et al. [2]	2016	To discuss the mechanism concerning the basis of ZIKV-induced neuropathogenesis.	Murine models to study ZIKV pathogenesis.	ZIKV tropism affects human neural progenitor cells (hNPCs).
Nayak et al. [12]	2016	To get an overview of the molecular mechanisms associated with ZIKV and its complications.	Studied several pathways that explain viral involvement in the brain and immune system.	ZIKV activates toll-like receptor 3 and induces the neurological complications associated with it.
Munoz et al. [21]	2016	To summarize the current evidence of ZIKV-related neurological complications.	Conducted ZIKV-related article search on Pubmed.	A direct viral effect on the neurons/glial cells is the pathogenetic mechanism of Zika-associated complications, including GBS.

Theory of Anti-Ganglioside in Zika-Associated GBS

GBS is an acute demyelinating disorder with a post-infectious origin. C. jejuni, Mycoplasma pneumoniae, Hemophilus influenzae, and viruses like influenza, Epstein-Barr, cytomegalovirus, dengue, chikungunya, Zika, and West-Nile have been associated with GBS. Autoimmunity between these microbial isotopes and human antigens is the primary mechanism of GBS pathogenesis. It is a form of ascending paralysis; hence it presents with lower extremity weakness and diminished deep tendon reflexes. Sensation presents with the loss of light touch, pain, and temperature in a “glove-and-stocking” pattern [23]. Here, we are focusing on the implications caused by ZIKV.

Dirlikov et al., in 2018, presented the postmortem results of a fatal GBS patient. They confirmed the presence of ZIKV by reverse transcriptase-polymerase chain reaction (RT-PCR) in urine, which also tested negative for all other organisms. Immunohistochemical staining of a section of cranial nerve IV showed myelin loss and abundance of macrophages. There was no evidence of direct tissue infection, suggesting antibody-mediated pathogenesis of GBS caused by ZIKV [7]. Sural nerve biopsies of patients infected with ZIKV showed a lack of the virus in the nerve tissue. Still, serum analysis in these patients showed circulating autoantibodies, which again confirmed the autoimmune mechanism of Zika-associated GBS [23]. In 2016, Unicini et al. presented a review that described the electrophysiological types of GBS that are associated with the ZIKV. Their results are consistent with acute inflammatory demyelinating polyneuropathy (AIDP) type, which affects the distal nerve terminals, which is absent in the blood-brain barrier and is more affected [24]. Describing the immunobiology of GBS, Willison, in 2005, used knock-out-mice to clone anti-ganglioside antibodies and induce the disease. He used a motor nerve terminal as the site of injury. Through this study, he proved the neuropathogenesis of murine anti-ganglioside antibodies and human GBS-associated antisera [25]. Such studies can be used to prove the autoimmune pathogenesis of GBS and to develop treatment strategies. To prove this point again in 2008, Willison and Plomp, used knock-out-mice to induce the autoimmune response between C. jejuni oligosaccharides and anti-ganglioside antibodies. They focused mainly on the axonal and glial components of neural tissue. The study results showed the presence of anti GM1/GD1a in acute motor axonal variant and anti-GQ1b/GT1a in Miller Fisher syndrome (MFS) [26].

Gangliosides are an essential component of nerve tissue. Anti-ganglioside antibodies are conjugated with FcγRIII receptor on macrophages to inhibit axonal regeneration [13]. At this point, the role of anti-ganglioside antibodies in the development of Zika-associated GBS was still unresolved. So, in 2018, Nico et al. tested the serum of patients affected with ZIKV for IgG autoantibodies against brain gangliosides by ELISA. They found a several-fold increase in the level of IgG autoantibodies to brain gangliosides in these patients [9]. Rivera-Correa et al. proved the same in 2019. They used the ELISA approach as well to assess the plasma of patients with ZIKV for their reactivity against different gangliosides. The assay results showed increased quantities of anti-ganglioside antibodies in patients with Zika-associated GBS than with patients who have Zika without GBS [1,8]. All these results show that the autoantibodies against gangliosides in a patient with GBS are associated with Zika too. The mechanism by which Zika elicits this immune response is by inhibition of RIG-1 like receptors, which are viral RNA sensors. They initiate an immune response by type I interferon production [27,28]. These findings [Table 2] provide us with a clearer understanding regarding the pathogenesis of Zika-associated GBS and the process by which the immune response is elicited.

**Table 2:** The studies used to explain the association of anti-ganglioside antibodies with Zika-associated GBS. TM: transverse myelitis, AIDP: acute inflammatory demyelinating polyneuropathy, GBS: Guillain-Barré syndrome, ZIKV: Zika virus.

Author	Year of Publication	Purpose of the Study	Intervention Studied	Result/Conclusion
Rivera-Correa et al. [1]	2019	To find the link between anti-ganglioside Ab and Zika-associated GBS.	Analyzed the anti-ganglioside Ab profile of plasma samples from patients with Zika infections in Salvador, Brazil.	The results established a link between anti-ganglioside antibodies and Zika-associated GBS in patients.
Dirlikov et al. [7]	2018	To analyze the postmortem findings of patients with Zika-associated GBS.	They performed autopsies on patients who died from GBS infected with Zika.	Results revealed demyelination of the sciatic and cranial IV nerves, which proved the association of ZIKV with AIDP type of GBS.
Rodrigez et al. [27]	2018	To focus on the putative role of infectious agents as triggering factors of GBS and TM.	N/A.	GBS related to infections frequently produce antibodies against human peripheral nerve gangliosides.
Acosta-Ampudia et al. [28]	2018	To show mechanisms underlying the development of autoimmune neurological conditions associated with ZIKV infection.	Analyzed patients with GBS who were infected with ZIKV.	IgG antibodies against GM1, GD1a, GalNAc-GD1a, and GM1b were found in patients with ZIKV-associated GBS.
Nico et al. [9]	2018	To find the prevalence of IgG antibodies against G3D gangliosides in acute ZIKV infection.	Studied patients with ZIKV infection and checked for the presence of GD3 antibodies.	The target of GD3 by autoimmune responses may affect the neuropathy and neurogenesis disorder seen during ZIKV infection.
Uncini et al. [24]	2016	To evaluate the literature on GBS association with ZIKV and other flaviviruses.	PubMed search to identify studies reporting GBS in association with ZIKV and other flaviviruses.	The electrodiagnosis of GBS in patients with ZIKV and other flavivirus is supportive of AIDP.
Asthana et al. [13]	2016	To dissect the role of anti-ganglioside antibody in GBS.	Injection of synthesized anti-ganglioside antibodies into mice to check for the immune response against peripheral nerves.	Results show the failure of nerve regeneration due to conjugation of anti-ganglioside antibodies to FcγRIII present on the circulating macrophages.
Willison and Plomp [26]	2008	To explore the antibody-mediated pathogenesis model of GBS.	Used knockout mice to develop murine neuropathy phenotypes mediated by anti-ganglioside antibodies.	A relationship has been established between anti-GM1/GD1a antibodies and the acute motor axonal variant and anti-GQ1b/GT1a antibodies and MFS.
Willison et al. [25]	2005	To highlight the immunobiological aspects of GBS.	Checked the neuropathogenic role for murine models anti-ganglioside antibodies and human GBS-associated antisera.	The presence of GM1, GD1a, and GT1a gangliosides proved molecular mimicry as a mechanism for GBS development.
Wright et al. [23]	2019	To present a case report and mini-review regarding the ZIKV-associated GBS and aseptic meningitis.	We performed a non-systematic mini-review using the PubMed database.	ZIKV-associated GBS presents with an accelerated clinical course compared to classic post-infectious GBS.
Van den Berg et al. [8]	2014	To review the clinical signs, pathogenesis, and diagnostic criteria of GBS.	They studied mouse models for diagnosis and treatment.	Anti-ganglioside antibodies were detected in patients with GBS underlying the pathogenesis of molecular mimicry.

## Conclusions

ZIKV outbreaks have caused a myriad of neurological complications like GBS, microcephaly, encephalitis, and myelitis. The neuroinvasive nature of this virus enables it to cause microcephaly, and neurotropism enhances its ability to cause GBS. Pregnant women affected by this virus in their first trimester can transmit the risk of microcephaly to their babies. Most of the studies used in this article are focused on the association between the ZIKV with GBS. Molecular mimicry between ZIKV and human epitopes is the main documented mechanism by which GBS is caused. One case-control study documented earlier clearly shows the presence of anti-ganglioside antibodies in patients with GBS with a recent history of ZIKV infection. This proves that the gangliosides on the nervous tissue are the target of the ZIKV to cause GBS as a complication. It takes a lag time of 7-10 days for this virus to cause these complications up to which it is difficult to detect these antibodies in the patient’s serum or urine. The ELISA test of the patient’s urine is the main diagnostic method to detect the presence of anti-ganglioside antibodies. But still, this hypothesis lacks enough supporting evidence. It needs to be explored more in the future to develop vaccinations and treatment options to prevent such life-threatening complications in the patients affected by this virus.
